# Polish pregnant women’s knowledge on early childhood caries prevention and oral hygiene in children

**DOI:** 10.1186/s12889-023-17604-5

**Published:** 2024-01-02

**Authors:** Katarzyna Domosławska-Żylińska, Magdalena Łopatek, Magdalena Krysińska-Pisarek, Paulina Wiśniewska

**Affiliations:** 1 Department of Education and Communication, National Institute of Public Health NIH – National Research Institute, 24 Chocimska St, Warsaw, 00-791 Poland; 2 Department of Communicable Diseases and Surveillance, National Institute of Public Health NIH – National Research Institute, 24 Chocimska St, Warsaw, 00-791 Poland

**Keywords:** Early childhood caries, Health education, Oral health promotion, Pregnant women, Children

## Abstract

**Background:**

Early childhood caries (ECC) is one of the most common chronic diseases among children. In Poland 86.9% of six-year-olds have ECC. One of the factors determining adherence to ECC prophylaxis and oral hygiene is mothers’ knowledge. The aim of this study was to assess the level of knowledge demonstrated by pregnant women about ECC prevention and oral hygiene, and to analyse the determinants of this knowledge.

**Methods:**

A quantitative survey was conducted using Computer Assisted Telephone Interview technique on a randomly selected representative sample of 1,000 women over the age of 18 in their second and third trimesters of pregnancy. The significance level was established at 0.05 and *p*-values were presented as: *p* < 0.05, *p* < 0.01 and *p* < 0.001.

**Results:**

The highest percentage of wrong or “I don’t know” answers were related to questions about: the number of free dental check-ups for children (76.8%), the date of the child’s first visit to the dentist (66.5%), the age when the child has mixed dentition (72.2%). Women with higher education had better knowledge than women with lower or secondary education. Women with good and very good financial situation showed a higher level of knowledge compared to women with average, bad and very bad financial situation.

**Conclusions:**

When developing prevention strategies and educational programs as part of prenatal care for women to reduce the incidence of ECC, it is important to take into account the identified areas that need support and specific target groups (mothers with lower socioeconomic status).

## Introduction

Early childhood caries (ECC) is one of the most prevalent chronic diseases among children, posing a major public health challenge [1, 2]. It is estimated that 46.2% of children worldwide have dental caries in primary teeth [1]. In Poland, the problem of ECC affects 41.4% of children at the age of 3 and 86.9% of 6-year-old children [2]. From the global point of view, the incidence of ECC in Poland is very high, as confirmed, for example, in a report by the Ministry of Health as part of the General Oral Health Monitoring [3]. Among the countries surveyed, Poland has the highest rate of ECC among six-year-old children [4].

ECC is defined as a “presence of 1 or more decayed (noncavitated or cavitated lesions), missing (due to caries), or filled tooth surfaces in any primary tooth in a child 71 months of age or younger. From ages 3 through 5, 1 or more cavitated, missing (due to caries), or filled smooth surfaces in primary maxillary anterior teeth” [5]. The main risk factors for ECC include microbiological (including carious microorganisms such as Streptococcus Mutans, Lactobacillus spp.), dietary (frequent consumption of sweetened foods), genetic (enamel defects), behavioural (poor oral hygiene, late start of tooth brushing by children, irregular brushing habits) and environmental (low socioeconomic status) factors [6]. The consequences of ECC include both the health and psychosocial aspects, such as a higher risk of carious lesions in permanent teeth, increased risk of hospitalization and emergency dental interventions, higher medical costs, absenteeism from school or loss of learning ability [7]. ECC can be prevented, for example by implementing preventive measures, such as: introducing proper hygiene habits (regular tooth brushing with toothpaste with the right amount of fluoride), dietary habits (limiting the intake of sugar-containing products), regular dental check-ups [8, 9].

One of the elements that influence adherence to ECC prevention and oral hygiene is the knowledge and attitude of parents, or caregivers [10]. Children acquire habits related to nutrition and oral hygiene from their parents. One example is a study by Baskarados et al. showing a strong relationship between caregivers’ oral health literacy and children’s oral health [11]. According to the recommendations of the American Academy of Paediatric Dentistry (AAPD) and the European Academy of Paediatric Dentistry (EAPD), the role of caregivers is to assist and perform good oral hygiene practices in children under the age of six [12]. However, the percentage of caregivers who do not supervise and assist their children during brushing is very high [13]. It has been observed that mothers have a key role in shaping good oral hygiene practices in children [14]. This particularly holds true for tooth brushing frequency. The more importance mothers attach to their own oral hygiene, the higher the frequency of brushing in their children [15]. As research shows, many mothers-to-be lack sufficient knowledge of the risk factors and importance of ECC prevention, due to, for example, inappropriate attitudes toward oral hygiene and lack of institutional support, as well as insufficient information from health professionals [13].

According to studies, there is a close relationship between the introduction of education for mothers (as early as in pregnancy) in terms of oral health promotion, and the application of ECC prevention principles, reduced incidence of ECC and S. mutans carrier-state in their children [16]. Therefore, ECC prevention strategies should begin with educating parents-to-be, especially mothers, given that pregnant women are a group that take a high interest in gaining knowledge and demonstrate a proactive approach to making changes in daily health behaviours [17].

Considering the scale of the ECC problem among Polish children and the importance of parents’ (especially mothers’) knowledge and attitudes in terms of ECC preventative behaviours, a study was conducted to assess the level of pregnant women’s knowledge of early childhood caries prevention (1), analyse factors influencing this knowledge (2), and identify areas of knowledge that need support in preventing early childhood caries and applying proper oral hygiene to children. The results of the survey can assist policy makers responsible for developing ECC prevention strategies to develop more effective programs, while dentists and other health professionals – to identify the necessary body of knowledge that should be spread to improve the effectiveness of children’s health and oral hygiene promotion.

## Methodology

The quantitative survey was conducted among a representative group of 1,000 pregnant women. The study used the CATI (Computer-Assisted Telephone Interviewing) method, while the selection of participants was random. The survey tool was an opinion poll panel. A sampling frame was used to initiate contact with respondents, which included a database of contact numbers, including both landline and mobile phone numbers operating in Poland. Sociodemographic data was verified using the survey’s inclusion (metric) questions. Respondents to the survey were qualified by their place of residence and whether this was their first pregnancy or a subsequent one. Women over the age of 18 who were in their second and third trimesters of pregnancy qualified for analysis. The survey participants were informed about possibility to terminate the study at any point. The survey met the guidelines for protecting individuals in terms of their security and privacy. The study participants gave verbal informed consent to participate in the study. Before the study began, they were informed of the purpose of the study, the anonymization of the data, the scientific nature of the result application, and the possibility of withdrawing from participation at any time. Data for the study were collected between October and November 2022.

### Research tools

The survey questionnaire consisted of two parts. The first part contained 12 questions on sociodemographic details. The second part contained 24 questions on the knowledge level regarding oral health and hygiene as well as ECC. Questions in the part assessing the knowledge level were adapted to the current guidelines of the Polish Society of Paediatric Dentistry, and possible answers were correct, incorrect and “I don’t know” [18]. The main areas of knowledge examined included: frequency of tooth brushing, tooth brushing time, use of fluoride toothpaste, transmission of cariogenic bacteria from mother to child, consumption of sugars, and dental check-ups.

The questionnaire was developed by the Institute’s professional staff. Then, it was subjected to a pilot study in which 5 professional employees participated, including 4 dentists specializing in public health and 5 people who were young mothers or pregnant women. Based on the information obtained from the pilot study, improvements were made to make the questionnaire more accessible and more valuable. Criteria used to assess the knowledge were: low level of knowledge (score 0 to 8); moderate level of knowledge (score 9 to 16); high level of knowledge (score 17 to 24).

### Statistics

The Chi-square test was used to determine the relationship of two categorical variables in the study. For the relationship between a quantitative variable and a qualitative variable, due to the lack of normal distribution among the quantitative variables studied, non-parametric Mann-Whitney U tests (for two groups) or Kruskal-Wallis tests (for three or more groups) were used. A significance level of 0.05 was adopted, with *p*-values presented as consecutive significance levels: *p* < 0.05, *p* < 0.01 and *p* < 0.001. All calculations were performed using the R software (version 4.0.0) and Microsoft Excel.

## Results

The study included 1,000 women over the 12th week of pregnancy. The average age of the respondents was 30, with the youngest 18 years old and the oldest 55 years old. The largest group fell within the 20–29 age range (46%). Most of the women had university education (51.4%) and lived in rural areas (38.3%). The majority of the respondents were economically active (75%) and married (67.3%). The condition of their oral cavity was rated as good or very good by 62% of the women, 45.6% declared that their dentition currently required treatment for caries, while past treatment for caries was declared by 79.7%. (Table 1.)

**Table 1:** Sociodemographic characteristics of study subjects, n = 1000; %)

		Woman n (%)
Age	≤ 19	1.2
	20–29	46.0
	30–39	44.1
	≥ 40	8.7
Accommodation	Countryside	38.3
	Town ≤ 200,000	21.7
	Town 200,000-500,000	31.2
	Town ≥ 500	8.8
Education	Elementary or junior high school	2.4
	Basic vocational	6.4
	Secondary or post-secondary	39.8
	Higher education	51.4
Employment	Employed (full-time or self-employed)	75.0
	Student	3.5
	Unemployed	5.9
	Pensioner/Retiree	0.6
	Household leader	15
Marital status	Married	67.3
	In an informal relationship	24.9
	Single	5.5
	Widowed	0.2
	Divorced	2.1
Self-assessment of material situation	Very bad	1.8
	Bad	6.4
	Average	51.2
	Good	35
	Very good	5.6
Self-assessment of overall health	Very bad	1.3
	Bad	1.5
	Average	29.7
	Good	57.2
	Very good	10.3
Self-assessment of oral health status	Very bad	1.3
	Bad	5.6
	Average	31.1
	Good	49.2
	Very good	12.8
Currently, your teeth require treatment for caries	Yes	45.6
	No	40.9
	I do not know	13.5
Treatment of teeth in the past due to caries	Yes	79.7
	No	18.0
	I do not know	2.3

Most of the women surveyed (59.5%) showed a relatively high level of knowledge regarding oral hygiene and ECC prevention. A moderate level of knowledge was observed in 38.2% of the respondents, while a low level was found in 2.3%. The greatest difficulties (in the form of wrong answers or “I don’t know” answers) were associated with questions about the number of free (funded by the National Health Fund) dental check-ups for children (76.8%), the date of the child’s first visit to the dentist (66.5%), the age when the child has mixed dentition (72.2%), and determining the age at which the child can brush their teeth independently (60.9%) (Table 2).

**Table 2:** Responses regarding knowledge of oral hygiene and ECC prevention (%); n = 1000

	Question	Correct answers	Incorrect answers	“I don’t know” answers	Sum of incorrect and “I don’t know” answers
1	How many dental check-ups for children per year are funded by the National Health Fund (NFZ)?	23.3	19.2	57.6	76.8
2	Up to what age does a child have primary teeth and permanent teeth side by side (so-called mixed dentition)?	27.8	47.5	24.7	72.2
3	When is it recommended to make the first visit to the dentist with the child?	33.5	53.3	13.2	66.5
4	At what age should a child’s manual skills be good enough to clean their teeth thoroughly on their own?	39.1	53.8	7.1	60.9
5	Who should use dental floss?	49.9	40.8	9.3	50.1
6	Does the diet of a pregnant woman affect the formation of teeth in a child?	53.8	17.8	28.4	46.2
7	How should a toothbrush be stored?	58.1	30.9	11.0	41.9
8	Does varnishing primary teeth help to protect against caries?	52.6	10.1	31.7	41.8
9	Should tooth brushing with fluoride toothpaste start as soon as the first primary tooth appears?	60.9	25.2	13.9	39.1
10	After which month of a child’s life should food and liquids not be given through a baby bottle with a teat?	64.1	20.6	15.3	35.9
11	Can carious bacteria be transferred from a parent to the child through saliva contact by, for example, kissing the child on the mouth, using the same cutlery during feeding or licking the pacifier?	68.5	11.9	19.6	31.5
12	Should an infant’s mouth be washed before the appearance of teeth?	71.8	11.5	16.7	28.2
13	Can permanent teeth become infected with carious bacteria from primary teeth?	72.2	7.1	20.7	27.8
14	When should dental floss be used?	79.3	17.3	3.4	20.7
15	Why is sugar in foods bad for teeth?	90.4	5.6	4.0	9.6
16	Should a child under the age of 6 and their parents use the same toothpaste?	91.0	5.6	3.4	9.0
17	What should be the main beverage for a child during the day?	91.4	7.0	1.6	8.6
18	How long should tooth brushing take?	91.5	7.4	1.1	8.5
19	Why is it important to treat primary teeth?	91.7	3.9	4.4	8.3
20	Does limiting sweetened foods in the diet help reduce the risk of caries?	92.5	3.8	3.7	7.5
21	Who should clean the tongue surface?	92.6	5.0	2.4	7.4
22	How often should children have their teeth checked at the dentist?	92.8	5.6	1.6	7.2
23	How often should a child brush their teeth?	95.5	4.40	0.1	4.41
24	How often should a child’s toothbrush be replaced with a new one?	95.5	4.40	0.1	4.41

### Respondents’ knowledge level vs. sociodemographic factors

The analysis of the survey results revealed statistically significant relationships between three sociodemographic factors, i.e. education level, financial situation and being a parent, and the respondents’ level of knowledge.

#### Education

The knowledge was statistically significantly higher (*p* < 0.001) in women with university education than in those with secondary education or lower (Table 3).

**Table 3:** Level of knowledge on oral hygiene and ECC prevention according to education (n = 1000)

		Education level n(%)			
Question	Answer	Lower or secondary	Higher	Chi^2^	*p*
What should be the main beverage for a child during the day?	Correct	429 (88.3)	485 (94.4)	11.82	< 0.01
	Incorrect	46 (9.5)	24 (4.7)		
	I don’t know	11 (2.3)	5 (1.0)		
Why is sugar in foods bad for teeth?	Correct	425 (87.4)	479 (93.2)	9.84	< 0.01
	Incorrect	37 (7.6)	19 (3.7)		
	I don’t know	24 (4.9)	16 (3.1)		
Can carious bacteria be transferred from a parent to the child through saliva contact by, for example, kissing the child on the mouth, using the same cutlery during feeding or licking the pacifier?	Correct	305 (62.8)	380 (73.9)	14.55	< 0.001
	Incorrect	67 (13.8)	52 (10.1)		
	I don’t know	114 (23.5)	82 (16.0)		
How should a toothbrush be stored?	Correct	249 (51.2)	332 (64.6)	23.46	< 0.001
	Incorrect	185 (38.1)	124 (24.1)		
	I don’t know	52 (10.7)	58 (11.3)		
Does the diet of a pregnant woman affect the formation of teeth in a child?	Correct	239 (49.2)	299 (58.2)	12.43	< 0.001
	Incorrect	106 (21.8)	72 (14.0)		
	I don’t know	141 (29.0)	143 (17.8)		
Does varnishing primary teeth help to protect against caries?	Correct	299 (47.1)	298 (57.8)	12.296	< 0.01
	Incorrect	59 (12.1)	42 (8.2)		
	I don’t know	198 (40.7)	175 (34.0)		

#### Financial situation

Women with good (answers good and very good) financial situation showed a higher level of knowledge (*p* < 0.01) on ECC prevention and oral hygiene of children, compared to women with moderate as well as bad (answers bad and very bad) financial situation (Table 4).

**Table 4:** The level of knowledge on oral hygiene and ECC prevention according to financial situation

		Financial situation n(%)				
Question	Answer	Bad	Mean	Good	Chi^2^	*p*
Who should clean the tongue surface?	Correct	67 (81.7)	479 (93.6)	380 (93.6)	16.52	< 0.01
	Incorrect	9 (11.0)	23 (4.5)	18 (4.4)		
	I don’t know	6 (7.3)	10 (2.0)	8 (2.0)		
Does limiting sweetened foods in the diet help reduce the risk of caries?	Correct	69 (84.1)	471 (92.0)	385 (94.8)	13.8	< 0.01
	Incorrect	5 (6.1)	23 (4.5)	10 (2.5)		
	I don’t know	8 (9.8)	18 (3.5)	11 (2.7)		
What should be the main beverage for a child during the day?	Correct	64 (78.0)	471 (92.0)	379 (93.3)	35.24	< 0.001
	Incorrect	11 (13.4)	37 (7.2)	22 (5.4)		
	I don’t know	7 (8.5)	4 (0.8)	5 (1.2)		
Why is sugar in foods bad for teeth?	Correct	64 (78.0)	462 (90.2)	378 (93.1)	18.03	< 0.01
	Incorrect	10 (12.2)	29 (5.7)	17 (4.2)		
	I don’t know	8 (9.8)	21 (4.1)	11 (2.7)		

#### Bearing a child

The results indicate that the fact of having children significantly (*p* > 0.001) influenced mothers’ knowledge of ECC prevention and children’s oral hygiene. Women with children had incomplete knowledge regarding the recommended teeth brushing duration and the use of the same toothpaste as parents by children under 6 years of age, compared to women without children. When it comes to the remaining questions regarding the effect of tooth varnishing on caries protection and the number of free dental check-ups available to children, women with children had better knowledge compared to women without children (Table 5).

**Table 5:** The level of knowledge on oral hygiene and ECC prevention according to the fact of being a parent

		Status as a parent n(%)			
Question	Answer	Has a child	Doesn’t have a child	Chi^2^	*p*
How long should tooth brushing take?	Correct	441 (88.2)	474 (94.8)	14.06	< 0.001
	Incorrect	51 (10.2)	23 (4.6)		
	I don’t know	8 (1.6)	3 (0.6)		
Should a child under the age of 6 and their parents use the same toothpaste?	Correct	411 (82.2)	469 (93.8)	11.62	< 0.01
	Incorrect	40 (8.0)	16 (3.2)		
	I don’t know	19 (3.8)	15 (3.0)		
Does varnishing primary teeth help to protect against caries?	Correct	290 (58.0)	236 (47.2)	23.405	< 0.001
	Incorrect	60 (12.0)	41 (8.2)		
	I don’t know	150 (30.0)	223 (44.6)		
How many dental check-ups for children per year are funded by the National Health Fund (NFZ)?	Correct	141 (28.2)	91 (18.2)	15.491	< 0.001
	Incorrect	97 (19.4)	95 (19.0)		
	I don’t know	262 (52.4)	314 (62.8)		

## Discussion

Based on the survey, it can be concluded that there is a need to improve pregnant women’s knowledge regarding the principles of children’s oral hygiene and prevention of early childhood caries. Similar results were obtained in a study conducted in Poland in 2015 by Gaszczyńska et al., which found that approximately 40% of pregnant women lack basic dental knowledge [19]. These results indicate that there has been no improvement in knowledge over the past 8 years. International studies also raise the issue of low knowledge of ECC prevention demonstrated by both pregnant women and young mothers [20, 21]. This is important, given that parents’ (especially mothers’) perceptions and knowledge of their children’s oral health affect the preventive dental care their children receive at home and the use of dental services [22].

The study identifies areas of knowledge that need special attention when planning educational interventions. As research has demonstrated, areas for improvement vary from society to society [23]. We observed that women in Poland had the greatest difficulty in determining the number of free check-ups for children per calendar year, the age of mixed dentition, the child’s first dental visit, the child’s manual skills to brush independently. In Poland, children and adolescents up to the age of 18 are entitled, for example, to a free medical check-up (granted 3 times per calendar year) or a dental check-up including oral hygiene instruction (1 time per calendar year) [24]. Despite the availability of free check-ups, 60% of Polish children aged 3 and about 25% of children aged 5 have never been to a dentist [4]. These data indicate that education relating to the availability of free dental check-ups for children is necessary, especially given that $1/3$ of parents cite the economic factor as a barrier to using dental services [25].

The Polish women surveyed also had trouble correctly determining the timing of the first visit to the dentist with their child. Studies analysing the effectiveness of early prophylactic dental visits indicate that these allow for early prevention and identification of dental diseases, and are associated with fewer visits for conservative treatment and related expenses in the first years of life [26]. Saikia et al. note that dental care systems should specifically promote “early first dental visits” when a child is 1 year old or when the first tooth appears [27]. Early dental advice and prevention can prevent the development of caries and improve the oral health of children, especially those whose caregivers would otherwise not be exposed to important information on best oral hygiene practices [28]. Awareness of mixed dentition is important in terms of oral health, as primary tooth caries is a risk factor for the development of permanent tooth caries [29]. On the other hand, tooth brushing is one of the more effective ways to achieve and maintain good oral hygiene. Effective tooth brushing depends on proper tooth brushing technique. Children’s ability to use a toothbrush varies depending on their age, but also on their manual skills [30]. According to recommendations, up to the age of 7–8 years, parents should brush their child’s teeth while teaching them to do it on their own as the child develops their manual skills. In later years, caregivers should correct their child’s tooth cleaning, help and control the quality of oral hygiene even up to the age of 10 [18].

The analysis of factors affecting the respondents’ level of knowledge showed a correlation between socioeconomic status (education and financial situation) or the fact of being a parent and the level of knowledge. In the present study, a better financial situation was associated with a higher level of knowledge of ECC prevention. Research emphasises that financial situation not only affects the knowledge level, but is also a factor that significantly influences a child’s oral health [31]. In addition, studies indicate that mothers’ education level directly determines their knowledge of ECC and affects the prevalence and severity of ECC [32]. Mothers with higher education and those who were economically active showed higher levels of knowledge of ECC prevention and were more involved in preventive measures for their children [33]. The results of the study confirm the need for educational interventions aimed especially at families with low socioeconomic status. This intervention can be effective in increasing parents’ knowledge and skills to help them recognize their child’s dental needs [34]. In contrast, a study by Nurit et al. found that despite higher education, mothers’ knowledge of their children’s oral health was incomplete [35]. In the study, a factor that significantly influenced the knowledge level of respondents was the fact of having children. Interestingly, women with children had incomplete knowledge regarding the recommended teeth brushing duration and the use of the same toothpaste as parents by children under 6 years of age, compared to women without children. Relationships between the number of children and mothers’ knowledge on ECC prevention have been reported, among others, in a study by Nurit et al. The level of oral health knowledge was significantly associated with the number of children in the family. Mothers of one child showed less knowledge than mothers of ≥ 3 children, which may indicate a learning curve over time. Contradictory results were demonstrated in a study by Marin Sousa Azevedo, where mothers who had three or more children were four times more likely to lack knowledge on the subject matter compared to mothers who had one child [36].

One of the advantages of the present study was that it was conducted on a representative large group of respondents, which yielded a high statistical power for analysis. Adapting the questionnaire to the current guidelines of the Polish Society of Paediatric Dentistry allowed collecting up-to-date data. This study has potential limitations. The data analysed were declarative data. The study focused only on examining the level of knowledge without including behavioural analysis. This is an area worth exploring in the next project. In the future, it would also be worthwhile to conduct a study to deepen the knowledge of the characteristics of women whose knowledge requires improving. This will allow a more precise educational strategy to be developed.

## Conclusions

In conclusion, according to research, mothers play a vital role in developing their children’s behaviours and habits, and are a source of fundamental information on oral care and health. The results of the present study showed that expectant mothers had significant gaps in their knowledge of what affects their children’s oral health. Identifying areas of particular concern (including availability of free dental care for children, timing of the first dental visit, etc.) and factors affecting knowledge (socioeconomic status, being a parent) can help develop better prevention strategies and educational programs as part of prenatal care for mothers and parents to reduce the incidence of ECC [20].

## Data Availability

All data generated or analysed during this study are included in this published article.
